# Martian biolith: A bioinspired regolith composite for closed-loop extraterrestrial manufacturing

**DOI:** 10.1371/journal.pone.0238606

**Published:** 2020-09-16

**Authors:** Ng Shiwei, Stylianos Dritsas, Javier G. Fernandez

**Affiliations:** 1 Engineering Product Development Department, Singapore University of Technology and Design, Singapore, Singapore; 2 Architecture and Sustainable Design Department, Singapore University of Technology and Design, Singapore, Singapore; Université de Pau et des Pays de l'Adour (UPPA), FRANCE

## Abstract

Given plans to revisit the lunar surface by the late 2020s and to take a crewed mission to Mars by the late 2030s, critical technologies must mature. In missions of extended duration, in situ resource utilization is necessary to both maximize scientific returns and minimize costs. While this present a significantly more complex challenge in the resource-starved environment of Mars, it is similar to the increasing need to develop resource-efficient and zero-waste ecosystems on Earth. Here, we make use of recent advances in the field of bioinspired chitinous manufacturing to develop a manufacturing technology to be used within the context of a minimal, artificial ecosystem that supports humans in a Martian environment.

## Introduction

With plans to revisit the lunar surface and eventually send a crewed mission to Mars, future space exploration missions are likely to involve an extended surface stay [1]. For such missions, or perhaps even settlements, survival requires meeting basic human needs while Earth-independent. With an emphasis on in situ resource utilization [2], a sustainable extraterrestrial settlement must be a resource-efficient, closed ecological system [3].

To minimize energy cost, Martian manufacturing strategies capitalize on the abundant inorganic components readily available in the regolith of the planet’s surface. However, these manufacturing methods are based on technologies developed for the bountiful paradigm of Earth and are commonly characterized by processes involving elevated temperature and pressure [4, 5], polymers with complex and dedicated biosynthesis [6], limited reclamation [7], and niche uses [8]. Since any resource obtained on Mars comes at an opportunity cost, the sustainable production of these materials must be contextualized in a Martian ecosystem. Towards this objective, nature presents successful strategies of life adapting to harsh environments. In biological organisms, rigid structures are formed by integrating inorganic filler procured from the environment at a low energy cost (e.g., calcium carbonate) and incorporated into an organic matrix (e.g., chitin) produced at a relatively high metabolic cost [9, 10]. Chitin is a paradigmatic example of an organic matrix of mineralized composites; it is the second most abundant organic polymer on Earth (after cellulose) and biology’s recurrent solution to forming structural components. Chitin is produced and metabolized by organisms across most biological kingdoms, including most heterotrophs used as bio-converters of organic matter [11].

For food production and other life support systems on Mars, early explorers will rely on other biological life [12, 13] and, due to its ubiquity, chitin will likely be part of any artificial ecosystem (example in S1 Fig) [14]. Insects specifically have been identified as a key part of a potential Martian settlement as a high-yield protein complement to fulfil the caloric requirements of a human crew [15] and to process agricultural and biological waste [16]. Still, despite the almost unavoidable presence of large amounts of chitinous polymers in human-centered cycles and their potential to feed engineered bacteria populations [17], these biopolymers have limited nutritional value in vertebrates [18]. Therefore, in contrast to all biomolecules used to produce bioplastics, the extraction of chitin does not hamper or compete with the food supply; rather, it is a byproduct of it.

Chitin in its most acetylated form (e.g., taken from arthropods and mollusks) is a mostly inert molecule with the same manufacturability issues as cellulose. Highly deacetylated chitin chains, usually referred to as chitosan (e.g., those taken from fungi), can be easily protonated in a weak acid, inducing intermolecular repulsion forces and enabling dissolution in aqueous solvents. Working with simple chemistry suitable for early Martian settlement, we produced Martian biolith using chitosan derived from arthropod cuticle (shrimp, 75–85% Degree of Deacetylation) via treatment with sodium hydroxide, a component obtainable on Mars through electrolytic hydrolysis [19]. Chitosan was dissolved in a low concentration of acetic acid (i.e., 1% v/v), the simplest carboxylic acid and a common byproduct in both aerobic and anaerobic fermentations, which is a vital process in a biotechnological strategy suited for Mars [20, 21]. The pH-based, simplified chemistry used here for the extraction and manufacture of chitosan requiring only water (available in the form of subsurface ice), sodium hydroxide, and acetic acid, can be further simplified if the polymer is obtained from an ecosystem involving fungi and, therefore, not requiring deacetylation/NaOH, or can be avoided completely by the use of enzymatic fractionation [11].

Here, we approach the problem of staying on Mars from a bioinspired perspective by replicating chitinous bioinspired manufacturing [22] developed for the production of sustainable manufacturing on Earth [11]. The resulting Martian biolith and its associated chemistry involve Martian regolith simulant, ubiquitous biomolecules, and water-based solvents that are easily integrated into any ecological cycle(s) and avoids the need for complex polymer synthesis, shipping of specialized equipment, or dedicated feedstock. We demonstrate how this material, produced and used with minimal energy requirements, retains the versatility of its biological counterparts, enabling the rapid manufacturing of objects ranging from basic tools to perhaps even rigid shelters.

## Results and discussions

**Fig 1:**
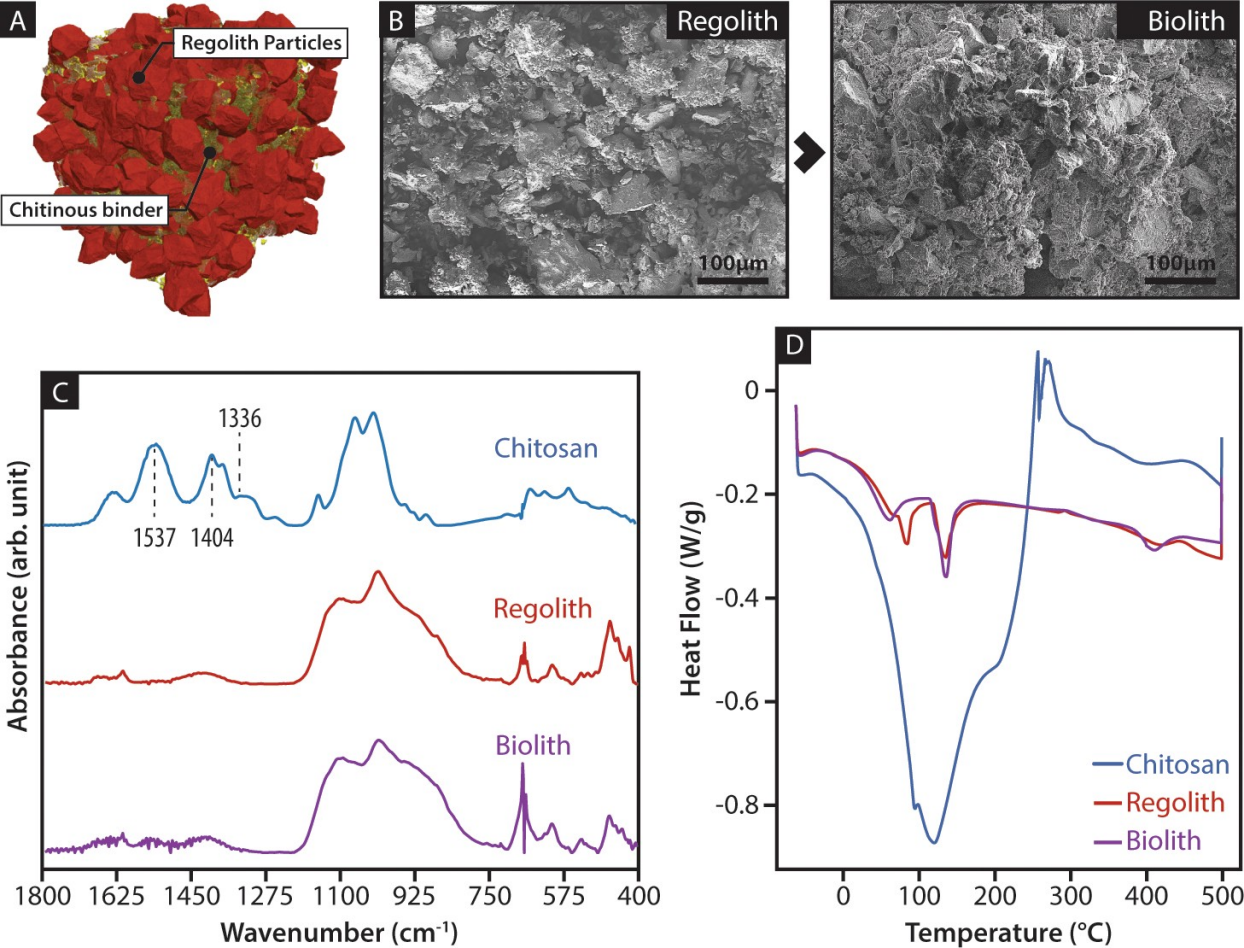
Micro-scale organization of biolith. (A) Regolith particles are dispersed within the chitosan solution after mixing. After the evaporation of the water, the chitosan crystalizes, reduces volume, and pulls the regolith particles together; (B) SEM imaging of regolith particle showing irregular size and morphology. Crystallized chitosan in the biolith can be seen enveloping and agglutinating the particles; (C) FTIR between chitosan, regolith, and biolith did not show conclusive evidence of a chemical reaction between chitosan and regolith; (D) Similarly, differential scanning calorimetry results did not reflect the heat flow patterns indicating phase changes between regolith and biolith.

**Fig 2:**
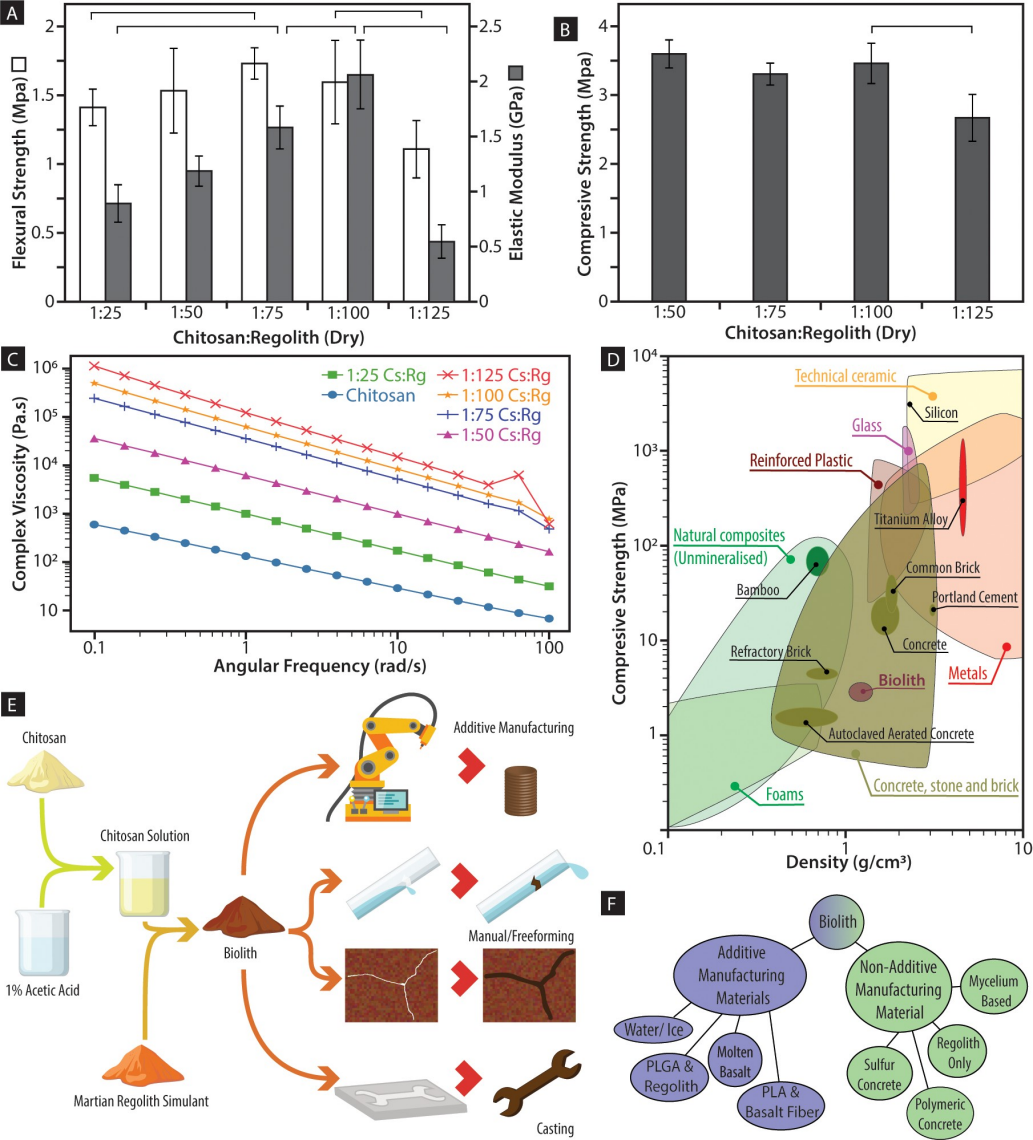
Mechanical characteristics of biolith composite. (A) Flexural strength and elastic modulus of biolith with varying chitosan to regolith ratio. There is a significant increase in flexural strength as regolith is increasingly added to a 1:75 ratio. At a 1:100 ratio, a significant decrease in flexural strength was observed. A similar relationship for elastic modulus was also observed; (B) A significant drop of compressive strength occurred beyond the 1:100 ratio; (C) Shear thinning behavior and increasing complex viscosity with added regolith was observed, likely due to the presence of chitosan; (D) Ashby plot showing dried biolith with mechanical properties similar to refractory brick; (E) Three different uses of biolith were demonstrated. From the prepolymer solution, the pliable liquid crystal-regolith mixture was cast into different geometries including a wrench that was later tested, used to repair a broken pipe, and used in additive manufacturing to produce a scaled habitat model; (F) The versatility to be shaped, printed, or casted without modification positions biolith as a unique material obtainable in a basic Martian ecosystem.

Chitosan forms transparent objects similar in appearance and mechanical characteristics to commodity plastics [23], a property lacking in the current materials deployable in early stage Mars settlements [7]. While chitosan on its own can be useful in specific applications, the composition of biolith (Fig 1A) has the minimum amount of metabolically expensive chitosan needed to produce a material with sound mechanical properties and general application. Three-percent chitosan in an acidic aqueous solution was used as external phase to form colloidal dispersions of Martian regolith simulant (Fig 1B) with weight ratios varying from 1:25 to 1:125, producing agglomerations of different bulk behaviors ranging from the inability to retain shape due to an excessive dispersive phase to the inability to form cohesive structures due to an excessive inorganic phase. Same trend was also observed and quantified in the mechanical testing of the material. Molecular and thermal analyses showed a lack of chemical interaction between the inorganic phase and the chitinous polymer (Fig 1C and 1D), suggesting that the composite is aggregated and internally compacted by strong intermolecular forces produced by the chitinous polymer during crystallization. After the aqueous solvent evaporated, the range of 1:75 to 1:100 led to the crystallization of the composite into solid structures with maximum flexural strength and stiffness (Fig 2A and 2B). These binder concentrations are between one-fifth and one-half of the amount of binder previously reported for “ultra-low-binder-content” composites (e.g., polystyrene (PS) or polylactic acid (PLA)) [24, 25].

**Fig 3:**
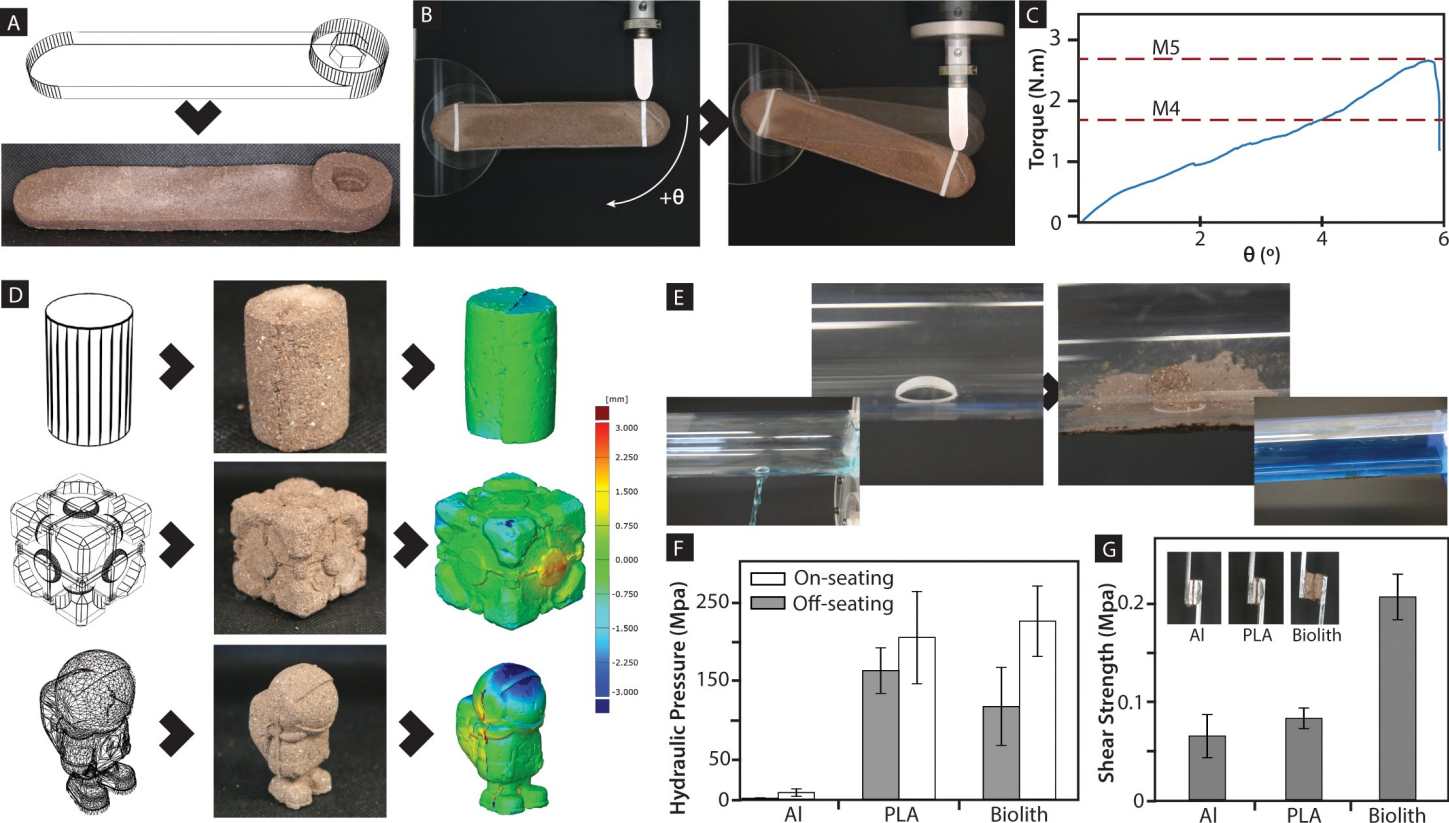
Demonstration of biolith utility in general manufacturing. (A) Custom-designed wrench casted with biolith that avoids failure at the handle; (B) The casted wrench was subjected to increasing vertical load after tightening to obtain maximum torque before failure; (C) The wrench sustained a maximum torque of more than 2.5 Nm before failure. In the absence of a torque wrench, the casted wrench can be designed to the desired failure load to prevent over tightening; (D) Molded samples of varying geometry intricacies to demonstrate biolith’s ability to replicate object geometries; (E) The ability of biolith to form a mechanical sealing is critical to stop leakage from a drilled hole in a chemically inert acrylic tube; (F) Differences in substrate material properties, such as material stiffness, could affect the maximum pressure sustained before the sealing is compromised via rupture or substantial leakage; (G) Initial hypothesis for enhanced sealing due to surface interaction was not validated as there was no significant difference in the shear strength of biolith applied onto aluminum or PLA surfaces.

The versatility of biolith in applications without elevated temperature or pressure (Fig 2C and 2D) is demonstrated in an unprecedented range of manufacturing methods, such as casting, using it as mortar and in additive manufacturing (Fig 2E and 2F). The initial products of biolith could be consumable tools and equipment mass fabricated by hand or casting. A pragmatic test for the casting potential of biolith was performed by producing a functional wrench (Fig 3A), which was tested by tightening a hexagonal bolt (Fig 3B). While biolith could not replace metallic tools, the simple wrench was able to sustain a torque of 2.83±0.92 Nm before breaking, matching the torque specified for M5 bolts used in non-critical space applications (Fig 3C) [26]. This demonstrates the practical utility of a tool made with biolith and its usefulness on Mars, as requirements would likely be different due to the lower gravity and atmospheric pressure. We also tested the ability of biolith to reproduce geometries via molding, from a simple cylinder to a more angular companion cube and, ultimately, a geometry involving both rounded and angular shapes (Fig 3D). The fidelity of these replicas was measured via comparative surface analysis using a high-resolution 3D scanner. While the average volumetric shrinkage among the three geometries was 9.9±3.6% due to the evaporation of water, the average surface deviation for the individual artifacts was small (-0.61±0.69 mm for the cylinder; -0.34±0.81 mm for the cube; -0.32 ±1.04 mm for the astronaut-like geometry).

Biolith could also be used as mortar for general maintenance purposes. Fig 3E shows biolith being used to fix a 12.5 mm diameter circular hole in a pipe. The elastic behavior of biolith enables a buckled internal plug to form and anchor the dried patch, producing a sealing effect based on mechanical principles rather than chemical bonding (i.e., like a rivet). No leakage was observed even after several weeks. The strength of the material in sustaining hydraulic pressure was measured using a modified burst test for biomaterials (ASTM F2392) under both negative and positive pressures (Fig 3F). In the more demanding operating condition that off-seats biolith sealing on precut biolith discs, the sealing was able to sustain pressures in the range of 117.70 ±49.67 MPa before the biolith sealing dislodged. For comparison, the atmospheric pressures on Earth and Mars are about 101.325 kPa and 0.61 kPa, respectively, while the Martian lander cabin pressure is likely to be around 56.53 kPa [27]. The hydraulic pressure sustained by the biolithic repairs of an aluminum disc (1.09±0.52 MPa) was significantly lower than that on the repairs of a synthetic polymers disc (PLA, 163.32±29.01 MPa) and the biolith disc. The difference was initially attributed to the different surface interactions between biolith and aluminum or PLA; however, this hypothesis was discarded due to the lack of a significant difference in bonding strengths between those materials (Fig 3G). This suggests that the low hydraulic pressure borne by biolithic repair on aluminum might be related to mechanical effects, such as a stiffness mismatch between the repaired part (aluminum) and the repair material (biolith).

**Fig 4:**
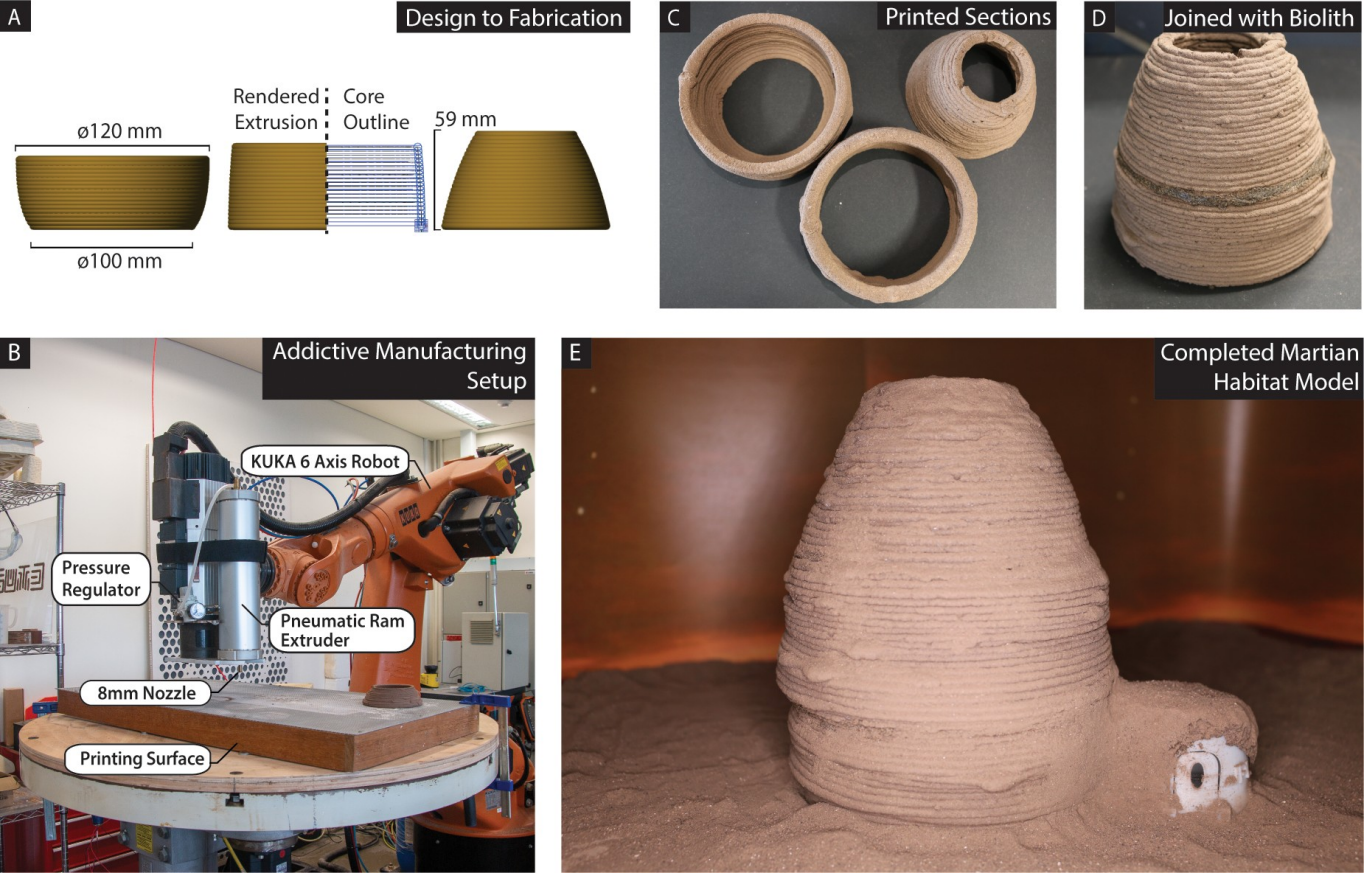
Usage of biolith in additive manufacturing. (A) A scaled model was created in three sections with varying degrees of overhang; (B) The additive manufacturing setup features a pneumatic ram extruder mounted onto a 6-axis robotic arm. Pressure is regulated to directly control the flow of material and used to match the translational speed of the robotic arm. Hot air is manually focused on extruded layers to accelerate water evaporation; (C) Printed sections after drying; (D) Sections joined using biolith as mortar; (E) Completed model with a 3D-printed lander module illustrating a possible scenario of fabricating habitats on Mars.

The self-adhesion of biolith is indicative of its suitability for additive manufacturing. For this purpose, a scaled ovoid replica inspired by the design of a NASA 3D printed habitat winner (MARSHA) was extruded in three segments and assembled using biolith (Fig 4A, 4C and 4D). The resulting structure was printed in 1.84 hours (Fig 4E). One of the advantages of the printing setup reported here (Fig 4B) is the ability to tune the printing process so as to balance speed and definition, as well as the ability to scale printed artifacts to several orders of magnitude using the same material and manufacturing technology. For example, using this technology, we printed a 5 m structure in 48h with a 5 mm definition, and its replica at 40 cm in 12 h with a 0.5 mm definition [11, 28].

## Conclusion

While scarce resources in an extraterrestrial environment pose extreme challenges to the establishment of a closed ecological cycle that supports human activities, it is conceptually similar to the problem of sustainable development on Earth. With past development based on the false premise of unlimited resources, we are facing the effects of a development model that is disconnected from Earth’s ecological cycles, resulting in both a shortage of primary resources and an accumulation of waste. Here, we applied the principles of bioinspired chitinous materials and manufacturing, initially developed for production within circular regional economies on Earth, to develop a composite with low manufacturing requirements, ecological integration, and versatile utility in a Martian environment. The results presented here demonstrate that the development of a closed-loop, zero-waste solutions to tackle unsustainable development on Earth may also be the key to our development as an interplanetary species.

## Materials and methods

### Materials

Chitosan (medium molecular weight; 75–85% deacetylated) used in the manufacturing process was purchased from i-CHESS chemicals Pvt Ltd., India. Glacial acetic acid was purchased from Sigma-Aldrich, Singapore. All chemicals were used as received. Mars Global Simulant, MGS-1, was purchased from The Exolith Lab, University of Central Florida and used as received except for additive manufacturing where regolith was sieved to limit regolith particles size to a maximum of 0.5mm.

Chitosan solution (3% w/w) was first prepared by dissolving chitosan in 1%(v/v) acetic acid. Next, biolith was prepared by manually mixing chitosan solution and regolith (MGS-1) in varying ratio (dry w/w) of 1:25, 1:50, 1:75, 1:100, 1:125.

### Methods

#### Additive manufacturing system

MGS-1 was sieved to limit regolith particles size to a maximum of 0.5mm in order to reduce the effects of phase and size separation during extrusion. Biolith used for additive manufacturing was prepared by mixing chitosan solution and sieved regolith (MGS-1) in 1:75 (dry w/w) ratio.

The core outline and robotic tool path of extrusion was generated in Rhino 3D modeler and translated into KUKA Robotic Language (KRL) through an algorithmic modeling plugin, Grasshopper. A movement command file would then be generated and uploaded onto KUKA KRC4 controller to control KUKA KR60HA. An endplate was machined to fit onto the end flange of an already attached electrospindle, ES929. A pneumatic tank, fitted with an 8mm nozzle, was mounted onto the endplate and pressurized by a pneumatic supply originally designed for changing tools on electrospindle. The rate of extrusion was directly controlled by a pressure regulator and set to be between 2 to 3 bars, above which would risk regolith compactions and phase separation. Phase and size separation during extrusion could be mitigated by exploring a different method of material delivery and possibly subject material to substantial shear forces only at point of extrusion. With extrusion of material at approximately constant flow rate, translational speed of robotic arm can be directly adjusted on the KRC4 pendant.

A hot air gun was used to direct warm air to accelerate biolith drying, slightly increase underlaying layer stiffness as extrusion continues and enhance buildability. Once extrusion is complete, the artefact is left for drying at room condition.

#### Casting of wrench and other geometries

In casting the wrench and geometries mentioned, silicone negative molds were made using positive 3D printed replicas. Biolith were prepared in 1:75 (dry w/w) ratios, manually fed into the molds and left to dry in room condition. They were further dried in a 60°C oven for 24 hours to remove excess moisture.

#### Pressure test and repair test

Repair test: Biolith were prepared in 1:75 (dry w/w) ratios and directly applied onto a 12.5mm hole drilled into an acrylic tube. The repaired pipe was left to dry in room condition and filled with water to observe leakage.

*Pressure test*. Biolith (1:75 ratio), aluminum and PLA discs of 55mm diameter and an internal hole diameter of 10mm was prepared. Wet biolith was applied onto individual discs to cap the internal hole and dried in ambient condition. The repaired discs were placed onto the base of test rigs with reusable putty at the bottom and top to surround and seal any leakages between the disk and rig interface. The test rig top was placed and secured with wing nuts. The peristaltic pump was switch on at a constant flow rate and burst pressure for each sample, whether physical dislodgement of biolith sealing or the inability to contain increasing pressure, was recorded.

#### Molecular characterization and imaging

Scanning electron microscopy: The surface morphology of dried biolith cross sections and as received regolith particles were examined using a Field Emission Scanning Electron Microscope (FESEM, JEOL, JSM-7600F). Samples were mounted onto a carbon tape on an aluminum stub and sputter coated with gold for 30 s.

Fourier-Transform Infrared Spectroscopy (FTIR) and Differential Scanning Calorimetry (DSC): For both experiments, biolith samples of 1:75 ratio (dry w/w) were dried and crushed. In addition, to better differentiate the observation of chitosan in biolith, 1%(v/v) acetic acid was added to both MGS-1 and dry chitosan powder in equivalent amounts as the biolith samples. This would make chitosan the differentiating factor in all three samples.

FTIR spectra were obtained using VERTEX 70 FTIR (Bruker optik GmbH), with a resolution of 4 cm−1 and accumulation of 32 scans between 4000 to 400 cm−1 on ATR mode. Heat flow measurements between temperatures of -60°C to 500°C were made using DSC Q20 (TA Instruments). Temperatures were ramped up at a rate of 10°C/min to 500°C, maintained at isothermal state for 2 mins and finally ramped down to -60°C at the same rate.

Rheological measurements: The flow behaviors of biolith were determined by conducting a frequency sweep from 0.1 to 100 rad on a Rheometer (HR-2 Discovery Hybrid Rheometer, TA instruments equipped with Environmental Test Chamber), using parallel plate geometry (40 mm diameter). Biolith with ratio 1:25, 1:50, 1:75, 1:100, 1:125 were prepared and immediately used for measurements. The experiments were conducted with a strain of 0.02 for all ratios except for 1:100 and 1:125 where a strain rate of 0.05 and 0.1 were used respectively.

#### Mechanical testing

Flexural Strength: Biolith with chitosan to regolith (dry w/w) ratio 1:25, 1:50, 1:75, 1:100, 1:125 were casted in a mold of 120mm by 6mm by 8mm for tensile test. The casted samples were dried in ambient conditions and further dried in an oven at 60°C for 24 h before testing using a UTM (Universal Testing Machine- Instron 5943) equipped with a 3-point flexural test fixtures. A support span of 99 mm and a strain rate of 0.01mm/mm/min was used. This test was a adapted from ASTM D790 (standard test method for Flexural Properties Of Unreinforced And Reinforced Plastics And Electrical Insulating Materials) procedure A for materials that breaks at relatively low deflection.

Compressive Strength: Biolith with chitosan to regolith (dry w/w) ratio 1:50, 1:75, 1:100, 1:125 were casted in a mold to form cubes of 15mm by 15mm by 15mm for compression test. The casted samples first dried in ambient conditions and further dried in an oven at 60°C for 24 h before testing till failure using a UTM (Universal Testing Machine- Instron 5943) equipped with parallel compression fixtures and at a speed of 1mm/min. No existing standard was used as finite regolith simulant limits small sample dimensions. Reference was made to a previous article where cubic samples of approximately 1 cm length was used.

Bulk adhesive Strength: A single lap shear test was conducted to evaluate biolith’s ability to adhere to Aluminum, PLA and biolith surfaces. The samples were prepared by first cutting a 3mm acrylic panel into 16mm by 100mm long strip and attaching square adherents of biolith, aluminum and PLA material by adhesives. With the individual adherents ready, wet biolith was applied over the 16mm by 16mm square adherents and another adherent was placed on top, secured with a bulldog clip and left for drying in ambient conditions. The samples were tested to on UTM following ASTM D3528 *tension loading testing for double lap shear adhesive joints* specifications, using 1 kN load cell at ambient condition and at a speed of 1.27mm/min.

Wrench failure test: An adjustable jig was made to accommodate a M10 hexagonal nut on which the wrench is secured. The initially free screw was gradually tightened using biolith wrench using hand until nut is sufficiently tighten and the tool was switched to a metallic wrench to fully tightened nut. The tool was switch back to the biolith wrench and used to produce further tightening torque to the already tightened nut to simulate the torque encountered by biolith wrench at a fully tightened nut. A downward displacement was created using a UTM (Universal Testing Machine- Instron 5943) equipped with a 3-point bending crosshead at a speed of 5mm/min. No standards and reference to other articles were used.

#### Statistical analysis

Experimental data were expressed as the mean ± standard deviation, with at least triplicate measurements. Hypothesis testing was done using Welch’s t-test with a two-tailed p value of 0.05.

## Supporting information

S1 Fig Illustration of a closed loop food production and waste management system on Mars and the use of chitin for in-situ manufacturing. (PDF)

S1 Table Dimensions of flexural samples. (PDF)

S2 Table Dimensions of compression samples (cubic). (PDF)

S1 Movie 3D printing of a segment of scaled model. Video is a time lapse of a 18 mins printing.
(MP4)

S2 Movie Casting of a wrench, wrench testing and pressure burst test. (MP4)
