# Supplementary material for: Martian biolith: A bioinspired regolith composite for closed-loop extraterrestrial manufacturing
Source: PLoS One. 2020 Sep 16;15(9):e0238606. doi: 10.1371/journal.pone.0238606 (PMC7494075; doi:10.1371/journal.pone.0238606)
Supplement: S1 Fig — (PDF) [file pone.0238606.s001.pdf]

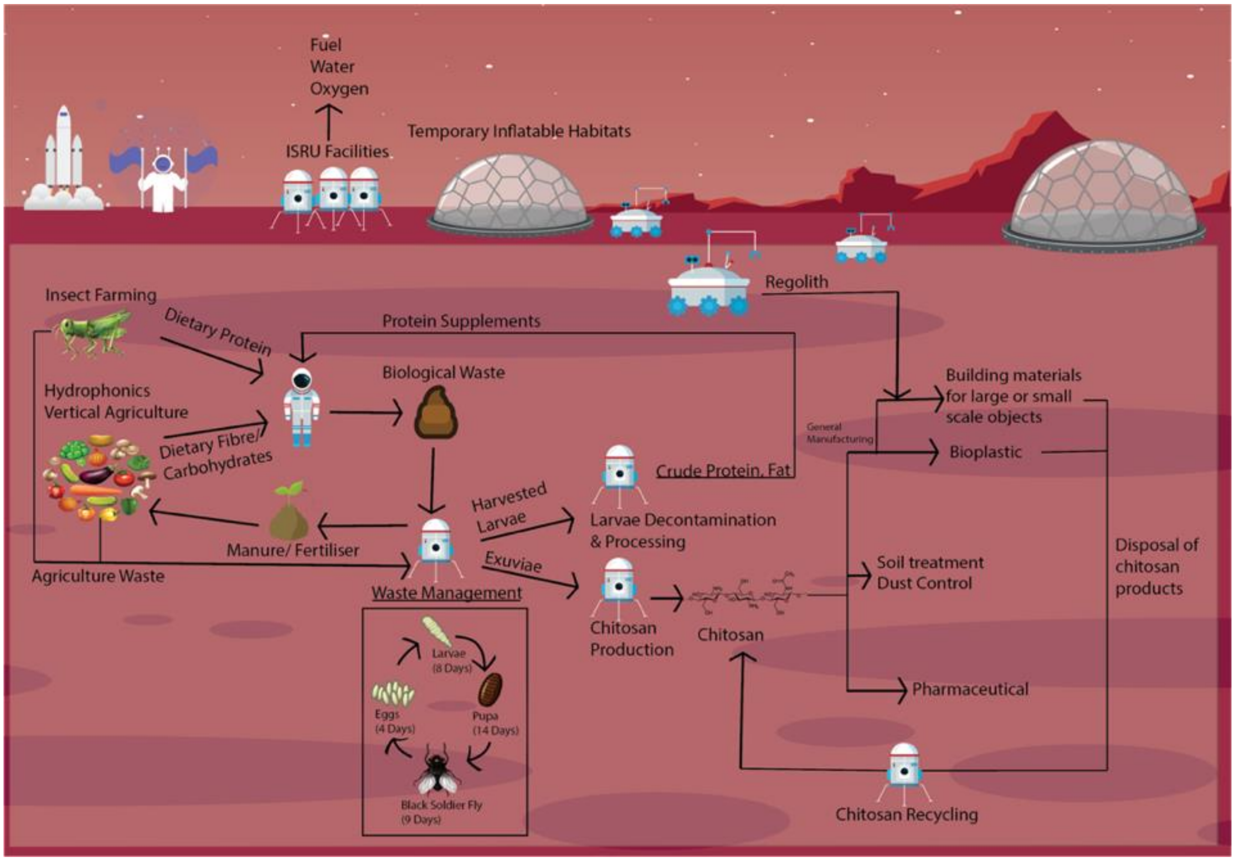

Fig S1. Illustration of a closed loop food production and waste management system on Mars and the use of chitin for in-situ manufacturing. Before arrival of human settlers, ISRU facilities producing necessities such as oxygen and water would be in operation and provides ready basic resources for human survival. To extend such stay, human settlers would need to build up local food production capacity and local waste management system. Both systems can be integrated into a closed loop system including humans and produces chitosan for manufacturing applications. Besides building small scale consumable tools or larger nonstructural shielding to prolong lifespan of inflatable habitats, chitosan can be used for a wide range of products including bioplastic tools (i.e. cups, films), pharmaceutical (wound dressings) and geotechnical application (soil stabilization, dust control). Considering the wide ranging application of chitin and its presence in a Martian ecosystem, the case for chitinous manufacturing on Mars holds much potential.
