# Supplementary material for: Martian biolith: A bioinspired regolith composite for closed-loop extraterrestrial manufacturing
Source: PLoS One. 2020 Sep 16;15(9):e0238606. doi: 10.1371/journal.pone.0238606 (PMC7494075; doi:10.1371/journal.pone.0238606)
Supplement: S1 Table — (PDF) [file pone.0238606.s002.pdf]

Table S1. Dimensions of flexural samples

Chitosan to Regolith (dry w/w ratio)	Width (mm)	Height (mm)	Length (mm)	Density (kg/m^3)	No. of samples
1:25	5.18 ± 0.33	6.16 ± 0.11	106.34 ± 0.96	1085.52 ± 58.40	6
1:50	5.40 ± 0.10	7.15 ± 0.09	112.11 ± 0.39	1264.74 ± 16.99	6
1:75	5.67 ± 0.06	7.65 ± 0.10	113.40 ± 0.39	1446.02 ±47.69	5
1:100	5.87 ± 0.25	7.90 ± 0.33	115.43 ± 0.14	1493.81 ± 62.40	6
1:125	6.17 ± 0.27	8.13 ± 0.17	116.44 ± 0.19	1596.07 ±41.89	6
