# Supplementary material for: Martian biolith: A bioinspired regolith composite for closed-loop extraterrestrial manufacturing
Source: PLoS One. 2020 Sep 16;15(9):e0238606. doi: 10.1371/journal.pone.0238606 (PMC7494075; doi:10.1371/journal.pone.0238606)
Supplement: S2 Table — (PDF) [file pone.0238606.s003.pdf]

Table S2. Dimensions of compression samples (cubic)

**Chitosan to Regolith (dry w/w ratio)**	**Side lengths (mm)**	**No. of samples**
1:50	12.13± 0.70	5
1:75	13.79± 0.14	5
1:100	14.36± 0.39	5
1:125	14.73± 0.22	5
